# [^18^F]BODIPY-triglyceride-containing chylomicron-like particles as an imaging agent for brown adipose tissue *in vivo*

**DOI:** 10.1038/s41598-019-39561-z

**Published:** 2019-02-25

**Authors:** Andreas Paulus, Natascha Drude, Emmani B. M. Nascimento, Eva M. Buhl, Jimmy F. P. Berbée, Patrick C. N. Rensen, Wouter D. van Marken Lichtenbelt, Felix M. Mottaghy, Matthias Bauwens

**Affiliations:** 1 Department of Radiology and Nuclear Medicine, NUTRIM School for Nutrition and Translational Research in Metabolism, Maastricht University Medical Center, Maastricht, The Netherlands; 2 Department of Nuclear Medicine, University Hospital RWTH Aachen, Aachen, Germany; 3 Department of Medical Imaging, Division of Nuclear Medicine, MUMC, Maastricht, The Netherlands; 4 Department of Nanomedicine and Theranostics, Institute for Experimental Molecular Imaging, Uniklinik RWTH Aachen and Helmholtz Institute for Biomedical Engineering, Aachen, Germany; 5 Department of Nutrition and Movement Sciences, NUTRIM School for Nutrition and Translational Research in Metabolism, Maastricht University Medical Center, Maastricht, The Netherlands; 6 Electron Microscopy Facility, Institute of Pathology, University Hospital RWTH Aachen, Aachen, Germany; 7 Department of Medicine, Division of Endocrinology, Leiden University Medical Center, Leiden, The Netherlands; 8 Einthoven Laboratory for Experimental Vascular Medicine, Leiden University Medical Center, Leiden, The Netherlands

## Abstract

Brown adipose tissue (BAT) is present in human adults and the current gold standard to visualize and quantify BAT is [^18^F]FDG PET-CT. However, this method fails to detect BAT under insulin-resistant conditions associated with ageing and weight gain, such as type 2 diabetes. The aim of this study was to develop a novel triglyceride-based tracer for BAT. For this purpose we designed a dual-modal fluorescent/PET fatty acid tracer based on commercially available BODIPY-FL-C_16_, which can be esterified to its correspondent triglyceride, radiolabeled and incorporated into pre-synthesized chylomicron-like particles. BODIPY-FL-C_16_ was coupled to 1,2-diolein with a subsequent radiolabeling step resulting in [^18^F]BODIPY-C_16_-triglyceride that was incorporated into chylomicron-like particles. Various quality control steps using fluorescent and radioactive methods were conducted before BAT visualization was tested in mice. Triglyceride synthesis, radiolabeling and subsequent incorporation into chylomicron-like particles was carried out in decent yields. This radiotracer appeared able to visualize BAT *in vivo*, and the uptake of the radiotracer was stimulated by cold exposure. The here reported method can be used to incorporate radiolabeled triglycerides into pre-synthesized chylomicron-like particles. Our approach is feasible to visualize and quantify the uptake of triglyceride-derived fatty acids by BAT.

## Introduction

**Figure 1:**
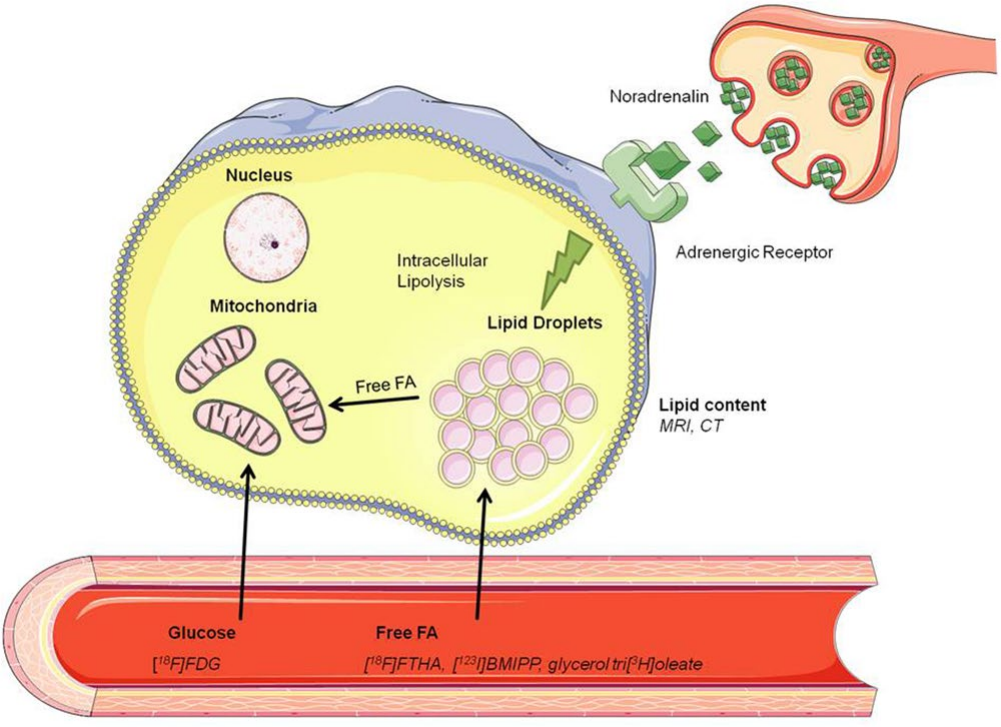
BAT activation by noradrenergic stimulation and possible quantification approaches by [^18^F]FDG (glucose consumption), [^18^F]FTHA, [^123^I]BMIPP, glycerol tri[^3^H]oleate-derived [^3^H]oleate (lipid uptake) and MRI, CT (lipid content).

Brown adipose tissue (BAT) research has evolved from an underestimated to a fast developing field in endocrine research and non-invasive imaging is an important technique to visualize and quantify BATs metabolic activity. Brown adipocytes have the ability to combust energy to heat by nonshivering thermogenesis in their mitochondria by virtue of the presence of uncoupling protein 1 (UCP1)^1^. It has been shown that BAT activation can be triggered by cold exposure, which induces a release of noradrenalin from nerve endings^2^. This neurotransmitter binds to adrenergic receptors on the BAT membrane and promotes an induction of intracellular lipolysis which leads to a release of fatty acids (FAs) from triglyceride (TG)-filled lipid droplets^3^ (Fig. 1). Those FAs activate UCP1 within the inner mitochondrial membrane^2^ whereby the proton gradient across the membrane is dispersed and heat is produced.

**Figure 2:**
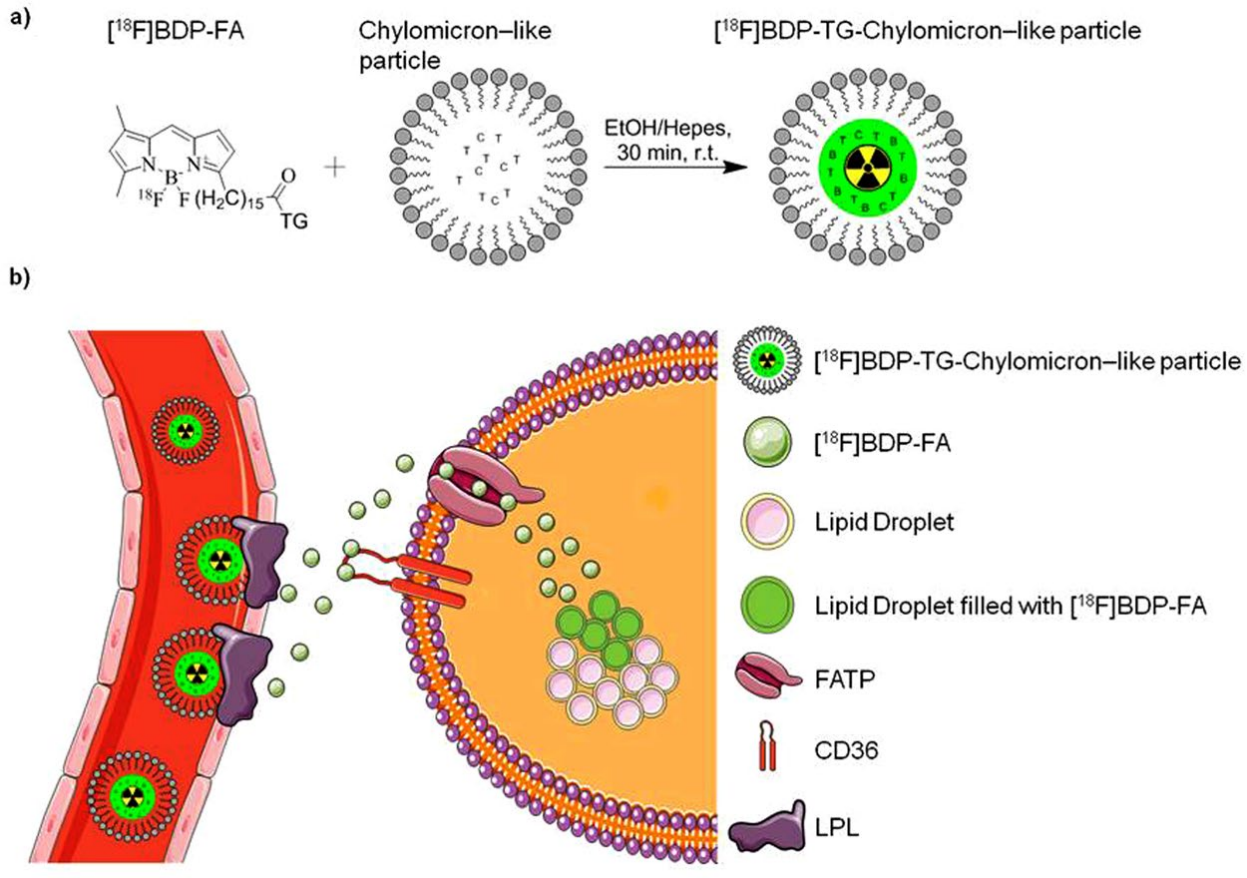
(**a**) Incorporation of radiolabeled [^18^F]BDP-TG into pre-synthesized chylomicron-like particles. (**b**) uptake of TG-rich lipoprotein (TRL)-derived FAs from the plasma facilitated by LPL, CD36 and FATP.

In activated BAT, internal lipid droplets are replenished by nutrient uptake from plasma in three different ways: uptake of TG-rich lipoprotein (TRL)-derived FAs, glucose uptake followed by *de novo* lipogenesis, and uptake of circulating albumin-bound FAs^2–5^. Although direct TRL particle uptake with adjacent FA release has been suggested^4^, more recent findings using glycerol tri[^3^H]oleate and [^14^C]cholesteryl oleate double-labeled TRL-like particles showed an approx. 10-fold higher uptake of FAs compared to cholesteryl esters by BAT^5,6^, indicating that the majority of TG-derived FAs is internalized after liberation by lipoprotein lipase (LPL). In fact, TRL-derived FAs were identified as the main supply of TGs in BAT^3,7^ and TG-derived FA internalization was shown to be dependent on the presence of lipoprotein lipase (LPL)^4^,^7,8^, cluster of differentiation 36 (CD36)^4,9^ and fatty acid transport proteins (FATP)^10^ (see also Fig. 2b).

The variety of quantification approaches of BAT volume and metabolic activity reaches from invasive imaging with fluorescence probes^4^ or ^3^H-labeled TG^5^ to non-invasive experiments with PET^11–15^, SPECT^16,17^, shorwave infrared^18^, targeted MRI with lipoproteins as contrast agent^4^, and MRI^19–21^ and was reviewed recently by us^22^. [^18^F]FDG (as a measure for glucose uptake) is frequently used in studies to quantify BAT activity and is currently the gold standard^12,23–25^ (Fig. 1). Nevertheless FAs are the main metabolized substance class in BAT, which is not simply mirrored by [^18^F]FDG scans^14^. In addition, insulin resistance such as type 2 diabetes of BAT occurring with ageing and weight gain will underestimate BAT activity as assessed with [^18^F]FDG scans, whereas FA and oxidative metabolism is not dependent on insulin sensitivity^26^. It should be mentioned that LPL activity was decreased during insulin resistance in mice^27^ and insulin was found to be essential for the lipolytic processing of TGLs by BAT^28^. Therefore BAT visualization with TRLs could become difficult during diabetic conditions, but it might be the most precise way to gain information about BATs lipid turnover and metabolic activity. Taken together, we reasoned a TG-derived FA tracer is needed to visualize and quantify lipid uptake by BAT to better reflect the thermogenic potential of BAT compared to [^18^F]FDG.

Radiolabeled FAs in general have been developed in several variations for imaging purposes (e.g. [^18^F]FTHA and [^125^I]BMIPP^29,30^). We here report the development of a FA-tracer based on fluorescent FA BODIPY-FL-C_16_ (BDP-FA), which is suitable for both PET and fluorescence imaging, and present a method to esterify the FA into a TG and subsequently incorporate it into a chylomicron-like particle. We hypothesized that by this approach the physiological situation is mimicked where TRL-derived TGs get lipolyzed locally before they are taken up by brown adipocytes. Additionally it will be possible to image from whole body to sub-cellular level and *in vitro* experiments can be carried out with the same molecule without any radiation dose. BODIPY dyes have been already used to image BAT^4^ and it is known that FA transport proteins (FATP) have a preference for BODIPY-FL coupled to a long carbon chain (C ≥ 8)^31^. Furthermore, downstream metabolic reactions in white and brown adipocytes have already been visualized *in vitro*^32,33^.

To avoid decreasing the FA characteristic properties of BDP-FA by introduction of another chelator molecule, we performed ^18^F/^19^F exchange reactions used to transform fluorescent dyes into dual-modality PET/fluorescent imaging dyes^4,34–37^. Neither an increased steric demand, nor lowering of the targeting efficiency of the FA towards proteins responsible for FA uptake is expected as BDP-FA is only modified at the end of the carbon chain and therefore keeps its FA characteristics. We recently described the synthesis and radiolabeling of [^18^F]BDP-TG and conducted first *in vitro* experiments with primary human adipocytes where [^18^F]BDP-FA uptake could be modified by different BAT activating and blocking agents^33^. The aim of the presented study was to incorporate the [^18^F]BDP-TG into chylomicron-like particles to obtain one of the first TG-based PET imaging agents and to test the new developed tracer in mice. Different to other used FA-based imaging tracers, the here presented TG will be first lipolyzed on-site before it can be internalized by BAT. This reflects the physiological situation in a better way than other FA-based tracers do and will help to quantify BATs consumption of lipids and its contribution to whole body energy expenditure.

## Results

### Synthesis of BDP-TG

Synthesis of BDP-TG was conducted as described before^33^. The resulting TG was obtained with a yield of 45 ± 8% SD after HPLC purification. BDP-TG has a t_r_ of 12.3 min and NMR and ESI-MS confirmed the identity of BDP-TG.

### Radiolabeling of BDP-TG

The radiolabeled dual-modality imaging agent [^18^F]BDP-TG was synthesized in a two-step procedure and was obtained with a decay corrected specific activity of 250 MBq/μmol and a decay corrected radiochemical yield of 44%. After washing with H_2_O [^18^F]BDP-TG was obtained with an overall decay corrected radiochemical yield of 21% and a radiochemical purity >96%. Shelf life and plasma stability showed 99% intact [^18^F]BDP-FA after 4 h^33^. Because of the insolubility of [^18^F]BDP-TG in aqueous medium shelf life and plasma stability experiments needed to be performed with [^18^F]BDP-FA, however no difference in stability is expected.

### Synthesis of chylomicron-like particles and characterization

Chylomicron-like particles were synthesized essentially as reported before^5,6,38^. After synthesis size of the particles was determined by DLS, showing a mean diameter of 164 ± 20 nm and a polydispersity index of 0.181 (n = 4). Additionally, particles were analyzed by transmission electron microscopy. Polydisperse particles could be visualized, all of which showed an encapsulated lipid core and a distinct surface shell with a mean particle diameter of 156 ± 55 nm (n = 25) (Fig. S1).

### *Ex vivo* incorporation of [^18^F]BDP-TG into chylomicron-like particles and characterization

**Figure 3:**
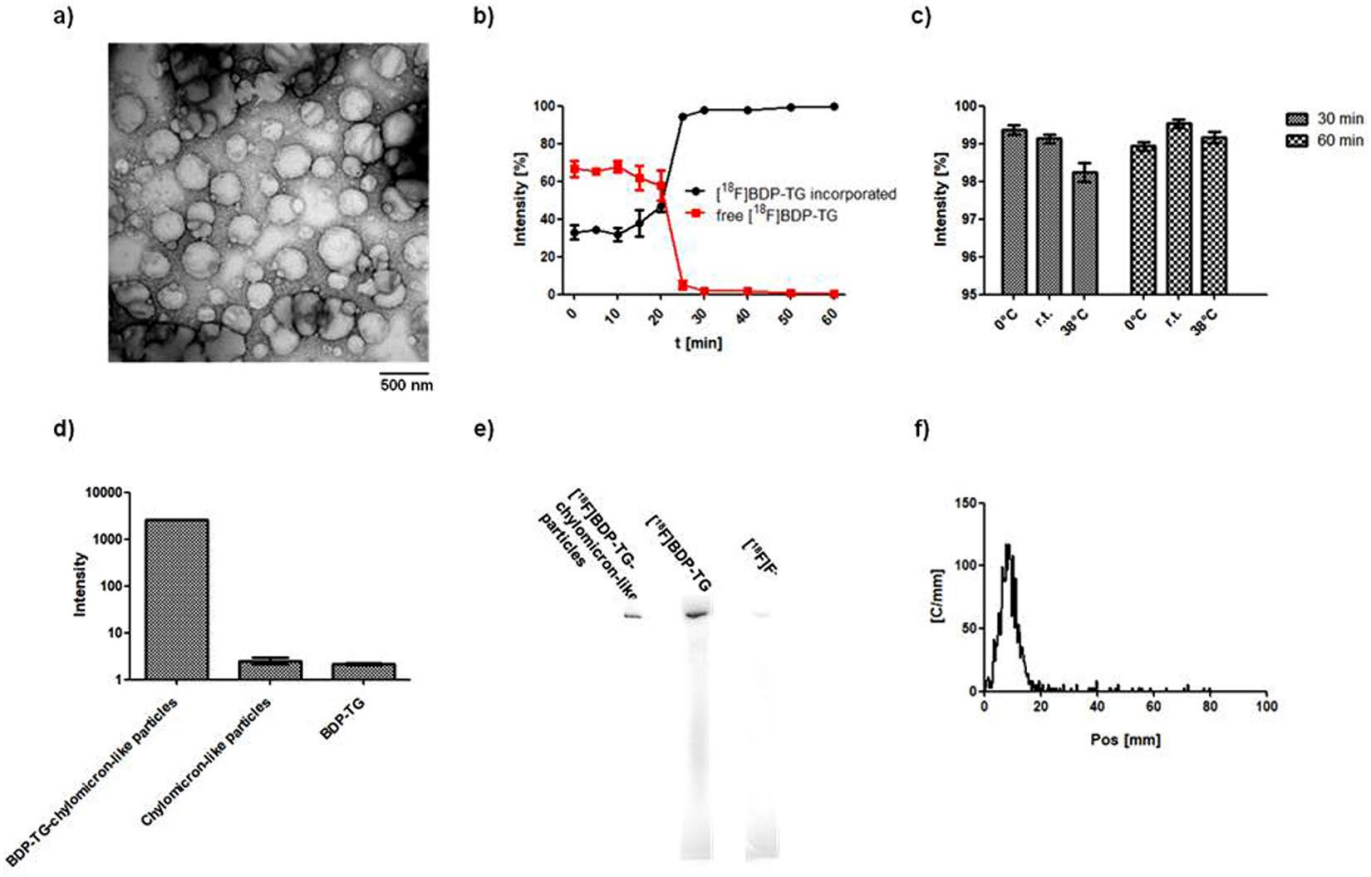
(**a**) Transmission electron microscopy of chylomicron-like particles incubated with [^18^F]BDP-TG. (**b**) Incorporation of [^18^F]BDP-TG into chylomicron-like particles. (**c**) Temperature dependence of the incorporation of [^18^F]BDP-TG into chylomicron-like particles. (**d**) Fluorescence measurement by a microplate reader of BDP-TG-chylomicron-like particles, chylomicron-like particles and BDP-TG. (**e**) Phosphorimaging after gel electrophoresis of chylomicron-like particles labeled with [^18^F]BDP-TG (starting position), [^18^F]BDP-TG (mainly at starting position) and free fluorine-18 (end position). (**f**) TLC of [^18^F]BDP-TG-chylomicron-like particles (Pos = 10 mm); possible impurities: free fluorine-18 (Pos = 10 mm), free [^18^F]BDP-TG (Pos = 70 mm).

After synthesis the chylomicron-like particles were loaded with BDP-TG or [^18^F]BDP-TG. Interestingly, once the BDP-TG is encapsulated, particles show a dark band within their lipid core (compare Fig. 3a with Fig. S1). Incorporation of [^18^F]BDP-TG into chylomicron-like particles in time was analyzed by TLC. After 25 min more than 99% of the TG was incorporated (Fig. 3b). Different temperatures did not affect the incorporation speed, where r.t. showed the best result after 60 min (99.5%) compared to 0 °C (98.9%) and 38 °C (99.1%) (Fig. 3c). To test whether the [^18^F]BDP-TG was truly incorporated, chylomicron-like particles loaded with BDP-TG were measured on a fluorescence microplate reader. Excitation was compared to chylomicron-like particles or BDP-TG alone. A significant increase in intensity (>1,400 fold) was observed for the chylomicron-like particles loaded with BDP-TG in comparison to the particles or the BDP-TG alone (Fig. 3d). Additionally, chylomicron-like particles incubated with [^18^F]BDP-TG did not show any impurities of free fluorine-18 or free [^18^F]BDP-TG after 60 min of incubation, analyzed by gel electrophoresis and radio-TLC (Fig. 3e,f).

### Animal experiments

**Figure 4:**
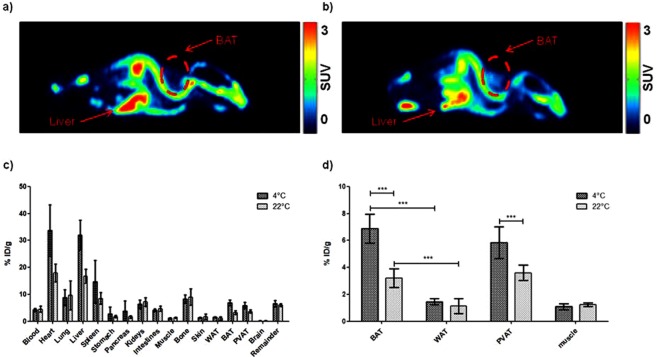
(**a**) PET image (40–60 min) of [^18^F]BDP-TG- chylomicron-like particles in a 22 °C fasted animal. (**b**) PET image (40–60 min) of [^18^F]BDP-TG-chylomicron-like particles in a 4 °C fasted animal. (**c**) Biodistribution of [^18^F]BDP-TG-chylomicron-like particles 1 h after injection. (**d**) BAT uptake in comparison to WAT, Aorta + PVAT and muscle.

[^18^F]BDP-TG-chylomicron-like particles (1–5 MBq) were injected into female C57Bl/6 mice, which were fasted for 4 h either at r.t. or at 4 °C. After scanning for 1 h the animals were euthanized and the organs were harvested. Analysis of the PET images showed highest uptake in liver and heart at r.t. and at 4 °C (Fig. 4a,b). A rapid increase with a slow washout in both organs could be visualized (Fig. S2a,b). In bone a constant increase in signal was observed (Fig. S2c,d), which probably indicates a defluorination process of the tracer *in vivo*, as reported in literature^37^. Lung showed a fast increase with a fast washout and stayed constant at later time points under both temperature conditions. Brain as a negative control showed negligible uptake. BAT uptake increased significantly when comparing cold exposed animals vs. control animals at 50–60 min p.i. (Mann-Whitney U, n = 10, two tailed p < 0.0001) (Fig. S2c,d).

PET images are supported by the results of the biodistribution. Highest uptake values in animals kept at r.t. were found in heart (17.9 ± 3.3% ID/g), liver (16.8 ± 2.6% ID/g), bone (9.0 ± 3.0% ID/g) and spleen (8.5 ± 2.2% ID/g). After 1 h only 4.5 ± 1.1% ID/g were found in the blood, indicating a fast blood clearance during the scanning time. In total 6.9 ± 1.3% ID was found in the blood calculated by an assumed total blood volume of 5.85 mL/100 g^39^. Uptake by BAT (3.5 ± 0.7% ID/g) was approximately 3-fold higher than uptake by WAT (1.1 ± 0.6% ID/g; Mann-Whitney U, n = 5, two tailed p < 0.001).

In fasted animals exposed to 4 °C highest uptake was reached in heart (33.7 ± 9.5% ID/g), liver (32.0 ± 5.5% ID/g) and spleen (14.7 ± 7.9% ID/g). Uptake in BAT was found to be 6.9 ± 1.1% ID/g and was significantly higher (Mann-Whitney U, n = 5, two tailed, p < 0.001) compared to WAT (1.5 ± 0.2% ID/g). Total activity remaining in blood was calculated to be 4.9 ± 0.8% ID and was therefore significantly lower (Mann-Whitney U, n = 10, two tailed, p < 0.01) compared to animals fasted at r.t. Organs which showed a significant difference between r.t. and cold exposed fasting were heart (Mann-Whitney U, n = 10, two tailed, p < 0.001), liver (Mann-Whitney U, n = 10, two tailed, p < 0.001), spleen (Mann-Whitney U, n = 10, two tailed, p < 0.01), BAT (Mann-Whitney U, n = 10, two tailed, p < 0.001) and perivascular adipose tissue (PVAT) (Mann-Whitney U, n = 10, two tailed, p < 0.001). No difference could be found in WAT, bone, muscle and all other analysed organs (Fig. 4c,d).

## Discussion

Exploring BAT and its metabolism has become an interesting and fast developing topic in endocrine research. A variety of different imaging approaches have been used in the past reaching from *in vitro* experiments^40^ over invasive imaging with fluorescence probes^4^ or ^3^H-compounds^5^ to non-invasive experiments with PET^11–15^, SPECT^16,17^ and MRI^19–21^. In a clinical environment most often [^18^F]FDG scans are used for BAT imaging but it only shows glucose-related uptake and has therefore the chance to misinterpret BAT activity by underestimating lipid uptake and metabolism. Additionally, [^18^F]FDG uptake is dependent on insulin sensitivity and therefore might not reflect the real activation state of BAT. In studies with [^18^F]FTHA it was observed that radiolabeled FAs showed an increased uptake in BAT under cold stimulation in humans^14^. However, these results with a free FA-based tracer might be less relevant because the majority of FAs is TRL derived^3,7^ where FAs are transported as TGs. With our developed tracer [^18^F]BDP-TG which is incorporated into chylomicron-like particles we can overcome these limitations and gain new insights in BATs lipid metabolism.

BDP-TG was produced in a decent yield (45%) like previously published^33^ and radiolabeling was carried out with a decay corrected radiochemical yield of 44% which is in accordance to radiolabeling yields reported in literature^36,41^.

Chylomicron-like particles have been synthesized with a mean diameter of 164 nm (DLS) and 156 nm (TEM). Those sizes are in accordance with previously described particles^5,6,38^.

After synthesis of the particles, loading with either BDP-TG or [^18^F]BDP-TG was performed. A pre-purification of [^18^F]BDP-TG from free fluorine-18 and SnCl_4_ before incubation with chylomicron-like particles is of immense importance. In a previous approach [^18^F]BDP-TG received a single wash with H_2_O (500 µL) before it was incubated with the particles. After 60 min the sample was purified by centrifugal filtration with an Amicon Ultra centrifugal filter (10 KDa). This caused two problems: 1) remaining SnCl_4_ caused a co-precipitation of the particles 2) filtration destroyed the particles yielding large lipid emulsions. This could be overcome by an intensified washing procedure where all the SnCl_4_ as well as free fluorine-18 was washed out before [^18^F]BDP-TG was added to the chylomicron-like particles.

An incorporation of the TG during the formation of the chylomicron-like particles in the sonicator has been tested but resulted in a breakdown of the boron-fluoride bond. Incorporation speed of [^18^F]BDP-TG into chylomicron-like particles was found to be fast (>99% after 60 min (Fig. 3b)) and temperature seemed to have no effect on the incorporation yield and speed (Fig. 3c). Purity of radiolabeled chylomicron-like particles was found to be >96% and therefore suitable for *in vivo* applications. (Fig. 3e,f).

Additionally, we evaluated the incorporation of the BDP-TG into chylomicron-like particles by fluorescence. We previously found that the fluorescence intensity is strongly related to the environment of the compound, meaning that only if the BDP-TG is dissolved it will give a fluorescence signal^33^. BDP-TG incorporated into chylomicron-like particles in HEPES solution showed a huge increase in signal (1413 fold) compared to BDP-TG or chylomicron-like particles alone. This indicates that once the BDP-TG is incorporated into a chylomicron-like particle it regains its fluorescence because it is in a lipophilic environment.

*In vivo* preferential uptake of [^18^F]BDP-TG in BAT compared to WAT was observed. Exposure to cold during fasting, thereby activating BAT^2,25^, pronounced the difference, indicating the ability of our tracer to visualize BATs metabolic activity and FA consumption. Although not visible on the microPET images, biodistribution data showed that PVAT followed a similar trend. Muscle tissue, which may become activated in the cold due to shivering^42^ showed only low uptake of [^18^F]BDP-TG and no increased uptake due to cold-exposure. This does not exclude a higher metabolic activity of muscle, as muscle may preferentially use glucose under conditions of cold exposure^43^.

BAT was however not the only tissue with a high uptake of [^18^F]BDP-TG. Uptake of the tracer was in fact highest in liver, heart, spleen and bone, and a significant increase by cold exposure is also demonstrated in liver, heart and spleen. Hepatocytes have a low LPL expression^6^. High uptake values can be explained by increased uptake of remnants of the chylomicron-like particles, which still contain TGs^44^, or by spill over of FAs generated during lipolysis^45^. This might also explain the elevated uptake due to cold activation because more remnants and FA are produced in this situation. Since the heart has high LPL expression^46,47^ a high tracer accumulation was expected. Increased LPL activity due to cold stimulation was already reported in cardiomyocytes^48,49^ which explains the increased heart uptake. The spleen, as an organ of the mononuclear phagocytic system, contains high numbers of macrophages. Those macrophages are able to engulf large particles which might explain the marked uptake observed in the spleen^50^. Lung uptake might be the result of a polydisperse particle distribution, as large particles are prone to get entrapped in lungs capillaries^51^. Indeed, in previous experiments with filtrated particles, where particle coagulation was frequent and sizes >1000 nm diameter could easily be reached, we experienced very high uptake values of >200% ID/g in the lung.

We also noted a high uptake of radioactivity in the bone. Although *ex vivo* plasma stability tests with [^18^F]BDP-FA showed >99% intact compound after 4 h^33^, this may not be valid *in vivo*. We were unable to demonstrate free fluorine-18 *in vivo* in the plasma, due to the rapid blood clearance and the difficulty to perform analyses on radiochemical purity of ^18^F-labeled chylomicrons from within a blood sample. Still, in recent literature it was shown that *in vivo* the stability is not ensured for radiolabeled BODIPY-compounds, making it likely that our compound is defluorinated as well^37^. In a former publication 1,3-diolein was coupled to BDP-FA^33^. We speculated that FAs on position 1 or 3 on the glycerol backbone might have a higher chance to get released during lipolysis. No significant differences in chemical and radiochemical yields during synthesis nor changes in the biodistribution can be reported for 1,3-diolein-BDP-FA vs. 1,2-diolein-BDP-FA (data not shown).

In comparison to our data, similar particles loaded with glycerol tri[^3^H]oleate previously showed higher uptake of [^3^H]oleate by BAT (approx. 5 fold^6^, 6 fold^5^ and 10 fold^52^) and lower [^3^H]oleate uptake by liver (approx. 0.6 fold^6^, 0.6 fold^5^ and 0.5 fold^52^) and heart (approx. 0.3 fold^6^, 0.3 fold^5^ and 0.5 fold^52^). Also no other organs showed an increased uptake when BAT was stimulated. Different experimental conditions (e.g. use of anesthetized vs. non-anesthetized mice) could be a reason for this difference in the biodistributions as gaseous anesthetics such as isoflurane are known to suppress adrenergic signaling^53^. In general these results may point to a reduced BAT LPL-activity due to anesthesia which results in increased uptake by liver and heart.

## Conclusion

In the current manuscript, we presented a dual-modal fluorescent and PET active TG which was successfully incorporated into chylomicron-like particles. With different quality control methods we showed incorporation of the radiolabeled TG into chylomicron-like particles. *In vivo* animal studies showed that the resulting tracer was able to reach BAT but was also taken up by other tissues which employ LPL-mediated FA uptake. BAT uptake of the tracer was increased in cold exposed animals. The here presented technique is able to visualize TRL-derived FA BAT uptake after TG-lipolysis which is advantageous in comparison to conventional FA-based tracers which do not reflect the physiological situation and are mainly taken up by the liver. We anticipate that [^18^F]BDP-TG-chylomicron-like particles are a promising step forward to visualize and quantify BATs lipid metabolism and gain more information about BATs contribution to whole body energy expenditure in the future.

## Methods

Commercially available compounds were used without further purification unless otherwise stated. BDP-FA was purchased from Thermo Fischer Scientific (99%) (Netherlands). 1,3-diolein was purchased from Sigma Aldrich (≥99%). 1,2-diolein was purchased from Cayman Chemicals (USA) (≥95%). DMEM/F-12 was purchased from ThermoFischer (Waltham, MA).

All HPLC purifications (1.0 mL/min, solvent A; 0.1% TFA in H_2_O, solvent B; CH_3_CN, 50 °C) were performed on a Shimadzu UFLC HPLC system equipped with a DGU-20A_5_ degasser, a SPD-M20A UV detector, a LC-20AT pump system, a CBM-20A communication BUS module, a CTO-20AC column oven, and a Scan-RAM radio-TLC/HPLC-detector from LabLogic using an Aeris™Widepore column (C4, 3.6 μm, 4.6 mm × 250 mm) for the BODIPY-triglyceride (BDP-TG). ESI-MS was performed on an Applied Biosystems SCIEX API 150 EX electrospray ionization quadrupole (ESI-Q) mass spectrometer with the method of McAnoy *et al*.^54^. Briefly, 0.1 M aqueous ammonium acetate solution was added to the sample to observe the ammonium salt of the synthesized TG in the MS.

^1^H-NMR spectra were carried out on a Bruker Ultrashield*TH 400 plus* at 400 MHz. Tol-d_8_ was used as solvent with TMS as internal standard. Chemical shifts are reported in parts per million (ppm) relative to the internal standard.

Gel electrophoresis was used to determine the amount of free fluorine-18 and [^18^F]BDP-TG in the solution containing [^18^F]BDP-TG incorporated in chylomicron-like particles. Gel electrophoresis was carried out under native running conditions where the sample was mixed (1:2) with native sample buffer and loaded into an any kD TGX gel (20 kBq per lane). For visualization phosphor screens were exposed for 10 h to the gel and analyzed by a Typhoon FLA 7000 phosphor imager (GE Healthcare).

### Synthesis of chylomicron-like particles

Synthesis of chylomicron-like particles was performed as reported before^38,55^. Briefly, emulsion particles were prepared from triolein (70 mg), egg yolk phosphatidylcholine (Lipoid) (22.7 mg), lysophosphatidylcholine (2.3 mg), cholesteryl oleate (3.0 mg), and cholesterol (2.0 mg). Sonification was performed using a Soniprep 150 (MSE Scientific Instruments, UK) that was equipped with a water bath for temperature (54 °C) maintenance, at 10 μm output. The emulsion was fractionated by density gradient ultracentrifugation steps in a Beckman SW 40 Ti rotor. After centrifugation for 30 min at 17,850 rpm at 20 °C, an emulsion fraction containing chylomicron-like particles was removed from the top of the tube by aspiration. Characterization of chylomicron-like particles was done by DLS and transmission electron microscopy. Chylomicron-like particles were stored at 4 °C and were used within 5 days following preparation.

### Dynamic Light Scattering

The particle sizes were measured by photon correlation spectroscopy performed at an angle of 90°; with a setup consisting of an ALV-SP8 goniometer, an ALV-SIPC photomultiplier, a multiple τ digital real-time ALV-7004 correlator, and a solid state laser (Koheras) with a red laser (λ = 633 nm) as light source. The time resolved signal of two Single Photon Counting Modules was cross-correlated. To prevent multiple scattering highly diluted chylomicron-like particle solutions of 0.1 mg/mL in bi-distilled and filtered H_2_O (1.2 μm poly(tetrafluoroethylene) membrane filters) were prepared. Sample cuvettes were immersed in a toluene bath and tempered within an error of ± 0.1 °C. Autocorrelation functions of intensity fluctuations g_2_ (q, t) are converted by the Siegert relation and give the field autocorrelation function f(q,t):

1 $$f(q,t)={\int }_{0}^{\infty }G({D}_{0})exp\{-{D}_{0}{q}^{2}\tau \}d{D}_{0}$$

Where τ is the decay time, G(D_0_) is the distribution function of D_0_, the diffusion coefficient and q as the scattering vector defined as

2 $$q=\frac{4\pi n}{{\lambda }_{0}}\,\sin (\frac{\theta }{2})$$

with θ being the scattering angle and λ_0_ being the wavelength of the laser light in vacuum.

The intensity-weighted decay-time τ distributions obtained from the field autocorrelation function by cumulant analysis were analyzed in respect to multimodality. For each diffusive mode the decay rate Γ = 1/τ was plotted against the squared length of the scattering vector q^2^. The slope gave the Z-average translational diffusion coefficient D_0_ and results in the hydrodynamic radius R_h_ after use of the Stokes Einstein equation:

3 $${D}_{0}=\frac{{k}_{B}T}{6\pi \eta {R}_{h}}$$

with q, k_B_, T and η being the scattering vector, the Boltzmann constant, absolute temperature, and solvent viscosity, respectively. A hydrodynamic radius distribution was calculated from the regularized Laplace inversion of correlation functions with CONTIN algorithm.

### Transmission electron microscopy

Samples were allowed to adsorb on glow discharged formvar-carbon-coated nickel grids (Maxtaform, 200 mesh, Plano, Wetzlar, Germany) for 3 min. Adhesive drops were removed by filter paper. Negative staining was performed with uranyl acetate (0.5% in H_2_O, Science Services GmbH, Munich, Germany) for 1–3 seconds. Excess liquid was removed, samples were air dried and examined using a LEO 906 E transmission electron microscope (Zeiss, Oberkochen, Germany), operated at an acceleration voltage of 60 kV.

### Synthesis of BDP-TG

Synthesis was performed as reported before^33^. Briefly, BDP-FL-C_16_ (300 μg, 0.6 μmol) in acetonitrile was evaporated to complete dryness before the reactant was reconstituted in toluene (100 μL). To the resulting solution SOCl_2_ in toluene (100 μL, 4 vol.-%) was added, incubated for 5 min at 70 °C in a closed vial and evaporated. The product was reconstituted in toluene (50 μL) containing 1,2-diolein (2 μL, 2.8 μmol) and heated to 100 °C for 30 min. After the reaction time, purification by HPLC (1 mL/min, 30% to 15% A in 5 min, 15% to 0% A from 5 to 6 min, 0% A to 20 min) yielded **2** (225 μg, 75%) as a red solid; t_R_ = 12.3 min. ESI-MS (+) m/z (%) = 1058 (100) [M − F^−^]^+^, 1095 (82) [M + NH_4_]^+^. ^1^H NMR (400 MHz, Tol-d_8_); δ (ppm) = 5.46 (m, 4 H), 4.26 (m, 2 H), 4.06 (m, 2 H), 3.13 (m, 1 H), 1.75 (s, 3 H).

### Radiolabeling of BDP-TG

Radiolabeling was performed as reported before^33^. Briefly, aqueous fluorine-18 solution was loaded on a QMA-cartridge which was preconditioned with 15 mL K_2_CO_3_ in H_2_O and 20 mL H_2_O. Fluoride (42 MBq) was eluted with a mixture of 600 μL acetonitrile, 400 μL H_2_O and 6 mg Sodium p-toluenesulfonate (Sigma-Aldrich). Fluorine-18 solution was transferred into a drying vessel containing tetra-n-butylammonium bromide (80 μL) as a phase transfer agent. Acetonitrile (3 × 1.0 mL) was added and the solution of fluorine-18 was dried by heating to 100 °C with a continuous flow of argon. After reconstitution of Fluorine-18 in anhydrous acetonitrile (100 μL), a solution of BDP-TG in toluene (107 μg, 0.1 μmol in 50 μL) and SnCl_4_ (0.2 M in acetonitrile, 100 μL) was added to the solution with the activity and the reaction solution was stirred at room temperature (r.t.) for 30 min. [^18^F]BDP-TG was obtained (decay corrected RCY: 44%, 25 MBq) with a decay corrected specific activity of 250 MBq/μmol and a radiochemical purity of 45% determined by a radio-TLC with toluene, CHCl_3_ and MeOH (80.9%, 14.3%, 4.8%) of the reaction solution.

### *Ex vivo* incorporation of [^18^F]BDP-TG into chylomicron-like particles

To [^18^F]BDP-TG (233 MBq) in the reaction solution 500 μL H_2_O were added and centrifuged for 5 min. The aqueous phase was aspirated and another 500 μL H_2_O were added to precipitate the remaining SnCl_4_. The mixture was heated to 100 °C, the organic phase was evaporated and the aqueous phase was taken off. [^18^F]BDP-TG was reconditioned in 20 μL EtOH and another radio – TLC was performed. [^18^F]BDP-TG could be obtained with a radiochemical purity of >96% and an overall decay corrected radiochemical yield of 21%. 400 μL chylomicron-like particles in HEPES were added (1.5 mg TG content) and incubated for 1 h at r.t. [^18^F]BDP-TG-chylomicron-like particles were obtained (overall decay corrected RCY: 18%, 19 MBq) with a radiochemical purity of >99% analyzed by gel electrophoresis and radio-TLC (Fig. 3e,f). Shorter incubation time points and different temperatures were tested by radio-TLC (Fig. 3b,c)

### Fluorescence measurements of BDP-TG-chylomicron-like particles

BDP-TG-chylomicron-like particles (80 μL) (0.1 μmol BDP-TG dissolved in 20 μL EtOH+ chylomicron-like particles (750 µg TG content in 200 µL HEPES), BDP-TG (80 μL) (0.1 μmol dissolved in 20 μL EtOH + 200 μL HEPES) and chylomicron-like particles (750 µg TG content in 200 µL HEPES) are measured using a SpectraMax M2 plate reader (molecular devices) (excitation 485 nm, emission 520 nm).

### Animal experiments

Experimental protocols were approved by the “Centrale Commissie Dierproeven” and all animal experiments and procedures were performed in accordance with the guidelines set of this institution. From 13:00 p.m. on, female C57Bl/6 mice (fasted for 4 h at r.t. or fasted and cold exposed at 4 °C for 4 h) were anesthetized (Isoflurane, 1.5–2.0% at 2 mL/min in oxygen), mice kept at r.t. were put on a heating pad and both groups were injected with [^18^F]BDP-TG-chylomicron-like particles (1–5 MBq) in HEPES (100 μL) via the tail vein. Mice were scanned dynamically for 1 h on a microPET (Focus 120, Siemens). Images were analyzed using Pmod V3.707. After the scanning time animals were killed and organs harvested, weighed wet and counted using a WIZARD^2^ automatic γ-counter from Perkin Elmer.

### Statistical analyses

Data are presented as mean ± SD, unless indicated otherwise. Differences at a probability level (p) of 0.05 were considered statistically significant. GraphPad Prism 5.01 (La Jolla, CA, USA) for Windows was used for statistical analyses.

## Supplementary information

Supplementary Information

## Data Availability

All data generated or analysed during this study are included in this published article (and its Supplementary Information files).

## References

[1] van Marken Lichtenbelt WD, Schrauwen P (2011). Implications of nonshivering thermogenesis for energy balance regulation in humans. American journal of physiology. Regulatory, integrative and comparative physiology.

[2] Cannon B, Nedergaard J (2004). Brown adipose tissue: function and physiological significance. Physiol Rev.

[3] Festuccia WT, Blanchard P-G, Deshaies Y (2011). Control of Brown Adipose Tissue Glucose and Lipid Metabolism by PPARγ. Front Endocrinol (Lausanne).

[4] Bartelt A (2011). Brown adipose tissue activity controls triglyceride clearance. Nat Med.

[5] Khedoe PPSJ (2015). Brown adipose tissue takes up plasma triglycerides mostly after lipolysis. J Lipid Res.

[6] Berbee JF (2015). Brown fat activation reduces hypercholesterolaemia and protects from atherosclerosis development. Nature communications.

[7] Hoeke G, Kooijman S, Boon MR, Rensen PC, Berbee JF (2016). Role of Brown Fat in Lipoprotein Metabolism and Atherosclerosis. Circulation research.

[8] Labbé SM (2015). *In vivo* measurement of energy substrate contribution to cold-induced brown adipose tissue thermogenesis. FASEB J.

[9] Coburn, C. T., Hajri, T., Ibrahimi, A. & Abumrad, N. A. Role of CD36 in membrane transport and utilization of long-chain fatty acids by different tissues. *J Mol Neurosci* **16**, 117–121; discussion 151–117, 10.1385/jmn:16:2-3:117 (2001). 10.1385/JMN:16:2-3:117 11478366

[10] Stahl A (2004). A current review of fatty acid transport proteins (SLC27). Pflugers Arch.

[11] Lee P, Greenfield JR, Ho KKY, Fulham MJ (2010). A critical appraisal of the prevalence and metabolic significance of brown adipose tissue in adult humans. Am J Physiol Endocrinol Metab.

[12] Cohade C, Mourtzikos KA, Wahl RL (2003). “USA-Fat”: prevalence is related to ambient outdoor temperature-evaluation with 18F-FDG PET/CT. J Nucl Med.

[13] Hany TF (2002). Brown adipose tissue: a factor to consider in symmetrical tracer uptake in the neck and upper chest region. Eur J Nucl Med Mol Imaging.

[14] Ouellet V (2012). Brown adipose tissue oxidative metabolism contributes to energy expenditure during acute cold exposure in humans. J Clin Invest.

[15] Bucci M (2015). Enhanced fatty acid uptake in visceral adipose tissue is not reversed by weight loss in obese individuals with the metabolic syndrome. Diabetologia.

[16] Syamsunarno MRAA (2014). Fatty acid binding protein 4 and 5 play a crucial role in thermogenesis under the conditions of fasting and cold stress. PLoS One.

[17] Putri M (2015). CD36 is indispensable for thermogenesis under conditions of fasting and cold stress. Biochem Biophys Res Commun.

[18] Bartelt, A. *et al.* Brown adipose tissue thermogenic adaptation requires Nrf1-mediated proteasomal activity. *Nature Medicine* **24**, 292–303 (2018). 10.1038/nm.4481 PMC5839993 29400713

[19] Grimpo K (2014). Brown adipose tissue dynamics in wild-type and UCP1-knockout mice: *in vivo* insights with magnetic resonance. J Lipid Res.

[20] Holstila M (2013). Measurement of brown adipose tissue mass using a novel dual-echo magnetic resonance imaging approach: a validation study. Metabolism.

[21] van Rooijen BD (2013). Imaging cold-activated brown adipose tissue using dynamic T2*-weighted magnetic resonance imaging and 2-deoxy-2-[18F]fluoro-D-glucose positron emission tomography. Invest Radiol.

[22] Paulus A, van Marken Lichtenbelt W, Mottaghy FM, Bauwens M (2017). Brown adipose tissue and lipid metabolism imaging. Methods (San Diego, Calif.).

[23] Cypess AM (2009). Identification and importance of brown adipose tissue in adult humans. N Engl J Med.

[24] Cypess AM (2015). Activation of human brown adipose tissue by a β3-adrenergic receptor agonist. Cell Metab.

[25] van Marken Lichtenbelt WD (2009). Cold-activated brown adipose tissue in healthy men. N Engl J Med.

[26] Blondin DP (2015). Selective Impairment of Glucose but Not Fatty Acid or Oxidative Metabolism in Brown Adipose Tissue of Subjects With Type 2 Diabetes. Diabetes.

[27] Qu S, Zhang T, Dong HH (2016). Effect of hepatic insulin expression on lipid metabolism in diabetic mice. Journal of diabetes.

[28] Heine M (2018). Lipolysis Triggers a Systemic Insulin Response Essential for Efficient Energy Replenishment of Activated Brown Adipose Tissue in Mice. Cell Metab.

[29] DeGrado TR, Coenen HH, Stocklin G (1991). 14(R,S)-[18F]fluoro-6-thia-heptadecanoic acid (FTHA): evaluation in mouse of a new probe of myocardial utilization of long chain fatty acids. J Nucl Med.

[30] Goodmen MM, Knapp FF, Elmaleh DR, Strauss HW (1984). New myocardial imaging agents: Synthesis of 15-(p-[123I]iodophenyl)-3(R,S)-methylpentadecanoic acid by decomposition of a 3,3-(1,5-pentanedyl)triazene precursor. J. Org. Chem.

[31] Dubikovskaya E, Chudnovskiy R, Karateev G, Park HM, Stahl A (2014). Measurement of long-chain fatty acid uptake into adipocytes. Methods Enzymol.

[32] Kasurinen J (1992). A novel fluorescent fatty acid, 5-methyl-BDY-3-dodecanoic acid, is a potential probe in lipid transport studies by incorporating selectively to lipid classes of BHK cells. Biochem Biophys Res Commun.

[33] Paulus A (2017). Synthesis, radiosynthesis and *in vitro* evaluation of 18F-Bodipy-C16/triglyceride as a dual modal imaging agent for brown adipose tissue. PLoS One.

[34] Liu S (2013). Lewis acid-assisted isotopic 18F-19F exchange in BODIPY dyes: facile generation of positron emission tomography/fluorescence dual modality agents for tumor imaging. Theranostics.

[35] Hendricks JA (2012). Synthesis of [18F]BODIPY: bifunctional reporter for hybrid optical/positron emission tomography imaging. Angew Chem Int Ed Engl.

[36] Keliher EJ, Klubnick JA, Reiner T, Mazitschek R, Weissleder R (2014). Efficient acid-catalyzed (18) F/(19) F fluoride exchange of BODIPY dyes. ChemMedChem.

[37] Paulus A (2015). Development of a clickable bimodal fluorescent/PET probe for *in vivo* imaging. EJNMMI Res.

[38] Rensen PCN (1995). Selective Liver Targeting of Antivirals by Recombinant Chylomicrons - a New Therapeutic Approach to Hepatitis-B. Nature Medicine.

[39] Bannerman, R. M. In *The Mouse in Biomedical Research* Vol. 3 *Normative Biology*, *Immunology*, *and* Husbandry (eds Small, J. D., Foster, H. L. & Fox, J. G.)293–312 (Academic Press, 1983).

[40] Henkin AH (2012). Real-time noninvasive imaging of fatty acid uptake *in vivo*. ACS Chem Biol.

[41] Liu S (2014). Efficient synthesis of fluorescent-PET probes based on [^18^F]BODIPY dye. Chemical communications (Cambridge, England).

[42] Gagnon DD (2014). The effects of cold exposure on leukocytes, hormones and cytokines during acute exercise in humans. PLoS One.

[43] Blondin DP (2015). Contributions of white and brown adipose tissues and skeletal muscles to acute cold-induced metabolic responses in healthy men. The Journal of physiology.

[44] Karpe F (2007). Removal of triacylglycerols from chylomicrons and VLDL by capillary beds: the basis of lipoprotein remnant formation. Biochemical Society transactions.

[45] Lewis GF, Carpentier A, Adeli K, Giacca A (2002). Disordered fat storage and mobilization in the pathogenesis of insulin resistance and type 2 diabetes. Endocrine reviews.

[46] Niu YG, Hauton D, Evans RD (2004). Utilization of triacylglycerol-rich lipoproteins by the working rat heart: routes of uptake and metabolic fates. The Journal of physiology.

[47] Bharadwaj KG (2010). Chylomicron- and VLDL-derived lipids enter the heart through different pathways: *in vivo* evidence for receptor- and non-receptor-mediated fatty acid uptake. J Biol Chem.

[48] Radomski MW, Orme T (1971). Response of lipoprotein lipase in various tissues to cold exposure. Am J Physiol.

[49] Keig P, Borensztajn J (1974). Regulation of rat heart lipoprotein lipase activity during cold exposure. Proceedings of the Society for Experimental Biology and Medicine. Society for Experimental Biology and Medicine (New York, N.Y.).

[50] Anselmo AC (2013). Delivering nanoparticles to lungs while avoiding liver and spleen through adsorption on red blood cells. ACS nano.

[51] Blanco E, Shen H, Ferrari M (2015). Principles of nanoparticle design for overcoming biological barriers to drug delivery. Nature biotechnology.

[52] Kooijman S (2014). Inhibition of the central melanocortin system decreases brown adipose tissue activity. J Lipid Res.

[53] Ohlson KB, Mohell N, Cannon B, Lindahl SG, Nedergaard J (1994). Thermogenesis in brown adipocytes is inhibited by volatile anesthetic agents. A factor contributing to hypothermia in infants?. Anesthesiology.

[54] McAnoy AM, Wu CC, Murphy RC (2005). Direct qualitative analysis of triacylglycerols by electrospray mass spectrometry using a linear ion trap. J Am Soc Mass Spectrom.

[55] Redgrave TG, Maranhao RC (1985). Metabolism of protein-free lipid emulsion models of chylomicrons in rats. Biochim Biophys Acta.

