# Supplementary material for: [18F]BODIPY-triglyceride-containing chylomicron-like particles as an imaging agent for brown adipose tissue in vivo
Source: Sci Rep. 2019 Feb 25;9:2706. doi: 10.1038/s41598-019-39561-z (PMC6389948; doi:10.1038/s41598-019-39561-z)
Supplement: Supplementary file 1 — Supplementary Information [file 41598_2019_39561_MOESM1_ESM.pdf]

[^{18}F]BODIPY-triglyceride-containing chylomicron-like particles as an imaging agent for brown adipose tissue *in vivo*

Andreas Paulus,[1,2,3] Natascha Drude,[2,4] Emmani B.M. Nascimento,[5] Eva M. Buhl,[6] Jimmy F.P. Berbée,[7,8] Patrick C.N. Rensen,[7,8] Wouter van Marken Lichtenbelt,[5] Felix M. Mottaghy,[2,3] Matthias Bauwens[2;*]

[1]Department of Radiology and Nuclear Medicine, NUTRIM School for Nutrition and Translational Research in Metabolism, Maastricht University Medical Center, Maastricht, the Netherlands

[2]Department of Nuclear Medicine, University Hospital RWTH Aachen, Aachen, Germany

[3]Department of Medical Imaging, Division of Nuclear Medicine, MUMC, Maastricht, The Netherlands

[4]Department of Nanomedicine and Theranostics, Institute for Experimental Molecular Imaging, Uniklinik RWTH Aachen and Helmholtz Institute for Biomedical Engineering, Aachen, Germany

[5]Department of Nutrition and Movement Sciences, NUTRIM School for Nutrition and Translational Research in Metabolism, Maastricht University Medical Center, Maastricht, The Netherlands

[6]Electron Microscopy Facility, Institute of Pathology, University Hospital RWTH Aachen, Aachen, Germany

[7]Department of Medicine, Division of Endocrinology, Leiden University Medical Center, Leiden, The Netherlands

[8]Einthoven Laboratory for Experimental Vascular Medicine, Leiden University Medical Center, Leiden, The Netherlands

Supplementary Information

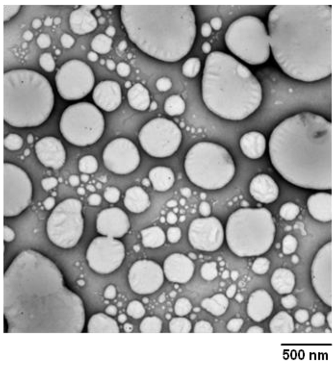

Fig S1: Representative picture of transmission electron microscopy of chylomicron-like particles without BDP-TG loading.

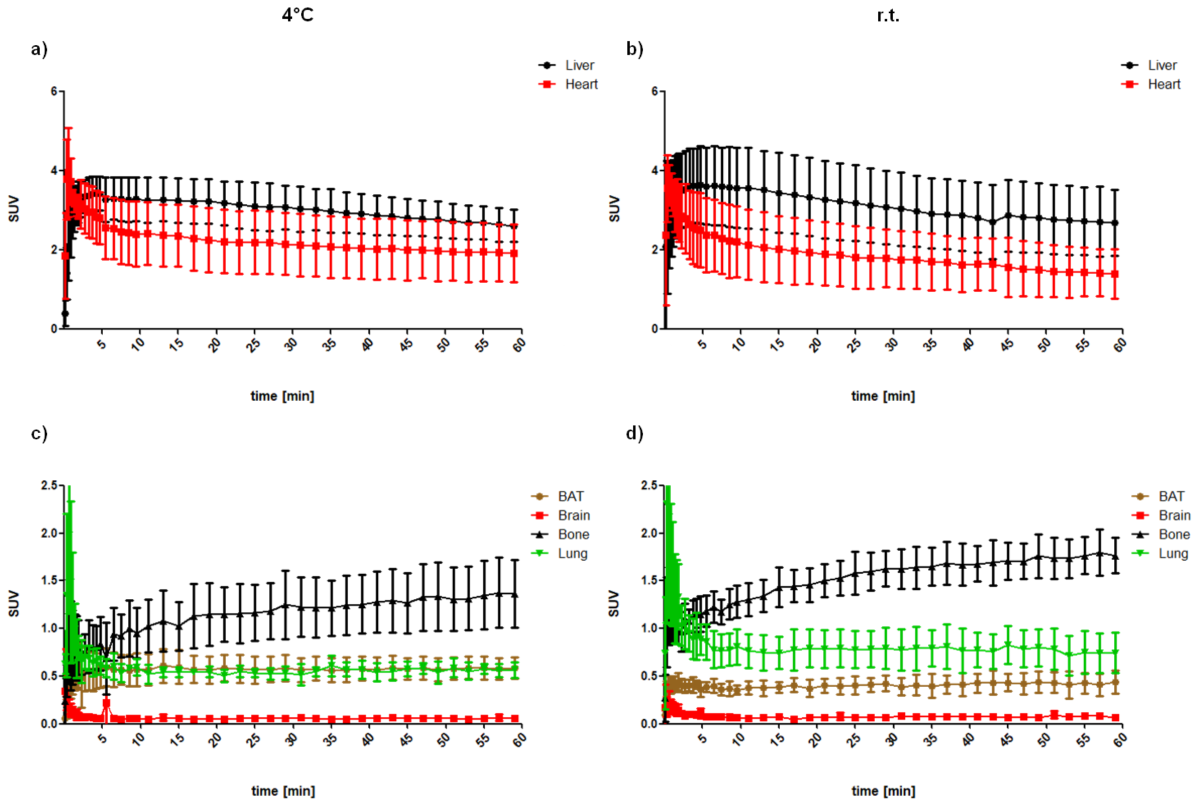

Fig S2: a) Time activity curves for specified organs in 4°C fasted animals b) Time activity curves for specified organs in r.t. fasted animals
